# The Consolidated Framework for Implementation Research: advancing implementation science through real-world applications, adaptations, and measurement

**DOI:** 10.1186/1748-5908-10-S1-A11

**Published:** 2015-08-20

**Authors:** Lucia Rojas Smith, Laura Damschroder, Cara C Lewis, Bryan Weiner

**Affiliations:** 1 RTI International, Washington, D.C., 20005, USA; 2 VA Ann Arbor Healthcare System, Center for Clinical Management Research, Ann Arbor, MI, 48105, USA; 3 University of Indiana, Department of Psychological and Brain Sciences, Bloomington, IN, 47405, USA; 4 University of North Carolina Chapel Hill, Gillings School of Global Public Health, Chapel Hill, NC, 27599, USA

## Introduction

The Consolidated Framework for Implementation Research (CFIR) draws together the unique and common elements of 19 different theories and frameworks and offers a common taxonomy for exploring the effectiveness of implementation within a specific context. Using the framework, the researcher or evaluator is able to address not only the question of "Does the intervention work" but more importantly for implementation and dissemination "For Whom?" and "Under what conditions?" Since its publication in 2009, the CFIR has been applied to 51 published syntheses or studies; a greater understanding of its current application will enhance its utility for future implementation research.

## Objective

The panel will present three applications and adaptations of the CFIR: (1) a synthesis of findings from six studies within the VA setting based on CFIR construct ratings from each; (2) the adaptation of CFIR to complex system interventions; and (3) results from a systematic review of quantitative measurement of CFIR constructs. This recent experience in using the CFIR in Implementation research suggests it can be a useful tool for assessing the complexity of healthcare transformation, particularly given the wide array of available quantitative instruments. The CFIR guides the user in considering the complex contexts of implementation in research and quality improvement, producing insights and results that can guide tailored implementation strategies. These strategies can be more systematically synthesized to inform the appropriateness, replicability, and sustainability of system transformation efforts to optimize care for patients. Together, through innovative methodologies (e.g., qualitative comparative analysis, enhanced systematic review), these projects will advance implementation science by identifying key contextual factors central to implementation success, delineating high quality measures for evaluating these factors and gaps in measurement, and highlighting an exemplary practical application of the CFIR to transforming complex systems.

## Source of funding

Work presented in this panel was supported by the Agency for Health Care Research and Quality (HHSA 290-2007-10056-I), National Institutes of Mental Health (R13; NIMH R13 MH086159-01A1) and the Department of Veteran Affairs, Health Services Research & Development Quality Enhancement Research Initiative (QUERI) RRP 12-494).

